# β-Blocker Use and Delayed Onset and Progression of Huntington Disease

**DOI:** 10.1001/jamaneurol.2024.4108

**Published:** 2024-12-02

**Authors:** Jordan L. Schultz, Amy C. Ogilvie, Lyndsay A. Harshman, Peg C. Nopoulos

**Affiliations:** 1 Department of Psychiatry, Carver College of Medicine at the University of Iowa, Iowa City; 2 Department of Neurology, Carver College of Medicine at the University of Iowa, Iowa City; 3 Department of Pharmacy Practice and Science, The University of Iowa College of Pharmacy, Iowa City; 4 Stead Family Department of Pediatrics at the University of Iowa, Iowa City

## Abstract

**Importance:**

Huntington disease (HD) is characterized by motor, cognitive, and psychiatric decline. β-Blockers may play a therapeutic role by decreasing enhanced sympathetic tone in HD.

**Objective:**

To evaluate the impact of β-blockers on the timing of motor diagnosis onset and progression of HD symptoms.

**Design, Setting, and Participants:**

This observational, longitudinal multicenter study used the Enroll-HD platform database (initiated September 2011 to present), including propensity score–matched cohorts of patients with premanifest HD (preHD) and early motor-manifest HD (mmHD) who were either users or nonusers of β-blockers. Participants included patients with genetically confirmed preHD (n = 4683 eligible participants) or mmHD (n = 3024 eligible participants) who were taking a β-blocker and were matched to similar non–β-blocker users.

**Exposure:**

Uninterrupted use of a β-blocker for more than 1 year.

**Main Outcomes and Measures:**

For PreHD: risk of receiving a motor diagnosis of HD over time. For mmHD: progression rate of total motor score, total functional capacity score, and the symbol digit modalities test. Post hoc analyses were performed to test additional clarifying hypotheses after the primary analyses were completed.

**Results:**

This study included 174 preHD β-blocker users (59 males; 115 females) with a mean age of 46.4 (SD, 13.1) years and a mean cytosine-adenine guanine repeat length of 41.1 (SD, 2.4) who were well matched to 174 preHD non–β-blocker users. The preHD β-blocker users showed a statistically significant reduction in the annualized hazard of receiving a motor diagnosis compared with nonusers (n = 174) (hazard ratio, 0.66; 95% CI, 0.46-0.94; *P* = .02). There were 149 mmHD β-blocker users (86 males; 60 females) with a mean age of 58.9 (SD, 11.3) years and a mean cytosine-adenine guanine repeat length of 42.0 (SD, 2.3) matched to 149 mmHD non–β-blocker users. The β-blocker users had a slower mean annualized worsening in total motor score (mean difference [MD], −0.45; 95% CI, −0.85 to −0.06; q = 0.025), total functional capacity score (MD, 0.10; 95% CI, 0.02-0.18; q = 0.025), and symbol digit modalities test (MD, 0.33; 95% CI, 0.10-0.56; q = 0.017) compared with matched nonusers.

**Conclusions and Relevance:**

In this study, β-blocker use was associated with delayed motor onset in preHD and reduced the rate of worsening of symptoms in mmHD. These findings demonstrated that β-blockers may have a therapeutic role in HD but further studies are required.

## Key Points

**Question:**

How does β-blocker use influence motor-diagnosis onset and progression rates in premanifest and early motor-manifest Huntington disease (HD)?

**Findings:**

In this study using the Enroll-HD platform, β-blocker users had a lower annualized hazard of motor diagnosis in premanifest HD and showed slower annualized worsening in motor and cognitive symptoms in early motor-manifest HD compared with nonusers.

**Meaning:**

These results demonstrated that β-blocker therapy in HD was associated with a later age at onset and a slower rate of worsening of clinical symptoms, suggesting a therapeutic potential for β-blockers in HD.

## Teaser

This study evaluates the impact of β-blockers on the timing of motor diagnosis onset and progression of Huntington disease symptoms.

## Introduction

Huntington disease (HD) is an inherited neurodegenerative disease caused by an expanded number of cytosine-adenine guanine (CAG) repeats within the *HTT* gene that encodes production of the Huntingtin protein.^1^ The disease is characterized by progressive motor, cognitive, and psychiatric decline.^2^ The number of CAG repeats within the *HTT* gene is inversely correlated with the age at onset (AO) of neurologic symptoms.^3^ CAG repeat length is also associated with the rate of progression of HD symptoms after the AO.^4^ However, environmental factors, including certain medications, are also associated with changes in both AO and in the rate of symptomatic progression of HD.^5,6,7,8,9^ Understanding modifiable factors that may impact AO and the rate of progression of HD may have implications in developing therapeutic agents in HD.

Patients with HD exhibit autonomic nervous system dysregulation, characterized by reduced parasympathetic and enhanced sympathetic tone.^10,11,12^ This can manifest as subtle, yet significant, elevations in heart rate and blood pressure.^10,13^ Recently, our group demonstrated that these changes in autonomic tone likely occur very early in life and are associated with decreased functional connectivity within the central autonomic network.^13^ This may support the notion that autonomic nervous system dysfunction is caused by pathologic changes in the brains of patients with HD. Consequently, the autonomic nervous system may be a novel target to help slow the progression of HD. β-Adrenergic receptor blockers (β-blockers) antagonize the action of norepinephrine at the β-adrenergic receptor. Norepinephrine is the primary neurotransmitter released by sympathetic neurons. Thus, β-blockers may uniquely block an overactive sympathetic nervous system in patients with HD. It is unclear if the use of β-blockers is associated with an altered disease course in patients with HD. To further investigate this possibility, we leveraged the Enroll-HD dataset to identify patients with premotor-manifest HD (preHD) and early motor-manifest HD (mmHD) who were using β-blockers to compare their disease courses relative to matched patients who were not using a β-blocker.

## Methods

Enroll-HD is a global research platform. Longitudinal data are collected from over 150 study sites around the world. We used the most recently available version of the Enroll-HD data, periodic dataset 6 (released November 4, 2022). We performed 2 primary analyses in distinct groups of participants with HD, which are outlined below. We followed the Reporting of Observational Studies in Epidemiology (STROBE) reporting guidelines.

### Participants

Enroll-HD enrolls individuals aged 18 years or older with genetically confirmed preHD and mmHD, plus at-risk individuals lacking genetic confirmation based on family history. Data collected by trained research personnel are transmitted to the data coordination center. Enroll-HD annually collects information on all research participants regarding current and past medications and data are scrutinized for quality and accuracy using a risk-based monitoring approach.^14^

We first identified patients with preHD, which was defined as patients with a diagnostic confidence level (DCL) less than 4 at their baseline visit.^15^ Additionally, participants had to have a CAG repeat length 36 or more but 55 or less to avoid including participants with juvenile-onset HD, who are known to have a significantly faster rate of progression relative to patients with adult-onset HD.^16^ Participants in the preHD group also had to have a baseline Unified Huntington’s Disease Rating Scale (UHDRS) total motor score (TMS) of 10 or less to increase the likelihood that they were, in fact, in the premotor-manifest stage of the disease. Only participants with preHD and data available from at least 2 study visits that were at least 1 year apart were considered for inclusion.

Next, we aimed to investigate the association between β-blocker use and the progression of symptoms in participants with mmHD. These participants also had to have a CAG repeat length between 36 and 55. Participants had to have a DCL of 4 during the Enroll-HD study and at least 1 additional visit (2 visits with a DCL of 4). Most participants with mmHD had a DCL of 4 at the time of their first available Enroll-HD visit; however, some participants received a motor diagnosis while participating in the Enroll-HD study and had subsequent visits with a DCL of 4. These participant visits were eligible for inclusion in the mmHD group. As a result, it was possible that a participants who received a motor diagnosis during the Enroll-HD study could have been included as a preHD participant and a mmHD participant in this study. Three participants were included as β-blocker users in both the preHD and mmHD analyses. mmHD participants also had to have data available that spanned at least 1 year to be included. In line with recent clinical trials in HD, we aimed to include patients who were relatively early in their HD disease course. We used guidance from the HD Integrated Staging System^17^ to identify patients with mmHD who only had mild diminishment of functional capacity. We included participants with a baseline independence scale score of 90% or more (eMethods in Supplement 1). Additionally, participants had to have a UHDRS TMS higher than 10 at baseline to increase the likelihood of including participants with early mmHD.

### Clinical Outcome Measures

For participants with mmHD, we used 3 separate outcome measures to compare clinical progression between β-blocker users and nonusers: (1) UHDRS TMS, (2) total functional capacity score (TFC), and (3) the symbol digit modalities test (SDMT). Further information on these measures are found in the eMethods in Supplement 1.

### Defining β-Blocker Users

β-Blocker users were those participants with a reported use of a medication with World Health Organization Anatomical Therapeutic Chemical Index numbers of C07AA or C07AB, C07AG, C07BA, C07BB, C07CB, and C07FB. Participants had to have been using 1 of these medications for at least 1 year at any time prior to the final visit or motor diagnosis to qualify as a β-blocker user (eMethods in Supplement 1).

For the analyses of participants with mmHD, participants were considered to be using a β-blocker if they began the medication of interest prior to their baseline visit. Given the longitudinal nature of this portion of the study, only visits where a participant was known to be taking a β-blocker were included for the β-blocker users. Therefore, if a participant discontinued therapy in between 2 of their annual visits, data from their upcoming visit would not be included in any analyses. This was done to avoid potential confounding that could emerge if there was significant time between a participant’s last dose of a β-blocker and their next visit.

The PreHD and mmHD groups included participants taking β-blockers and other antihypertensives, like angiotensin-converting enzyme inhibitors (ACEIs), angiotensin receptor blockers (ARBs), or thiazide diuretics. β-Blocker nonusers were participants who had either never used a β-blocker, had used a β-blocker for less than 365 days, or began using a β-blocker after the time interval of interest. There were 7 nonusers in the preHD group who used a β-blocker for less than 1 year.

### Statistical Analysis

#### Matching Procedures

We matched β-blocker users and nonusers using propensity score matching with the ‘MatchIt’ library.^18^ We performed 1-to-1 nearest-neighbor matching with a 0.1 caliper. For the primary analyses, 2 separate matches were performed. The first was conducted in participants with preHD while the second was performed in participants with mmHD. The matching procedures for the participants with preHD and mmHD differed. Specifically, we selected participants with preHD with minimal clinical symptoms to ensure we were assessing participants in the preHD stage of the disease. Due to the lack of significant clinical symptoms at baseline, participants were not matched on baseline clinical symptoms. The participants with preHD were matched on their baseline age, CAG repeat length, sex as a biological factor, baseline body mass index, history of tobacco use, diagnosis of hypertension and/or diagnosis of type 2 diabetes, and use of a statin. In contrast, participants with mmHD were matched on the above characteristics, as well as baseline TMS, baseline TFC, and baseline SDMT score to account for differing clinical symptoms. Participants reporting a diagnosis of hypertension or type 2 diabetes at any point were classified accordingly. Statin use was determined similarly. Analyses used RStudio version 1.3.159 (R Project for Statistical Computing) with significance set at *P* < .05 or *Q* < 0.05.

#### Survival Analysis in Participants With preHD

We compared the annualized risk of receiving a motor diagnosis between β-blocker users and nonusers using a Cox regression survival analysis. This analysis was doubly robust in that all variables that participants were matched on (see Matching Procedures) were also included as covariates. For this analysis, survival was considered the number of years from the baseline visit until the participants received a motor diagnosis, defined as the first DCL of 4. Participants who did not receive a motor diagnosis were indicated as having remained in the preHD phase of the disease at the time of their last known Enroll-HD visit.

#### Longitudinal Analysis in Participants With mmHD

After matching mmHD β-blocker users to nonusers, we constructed linear mixed-effects regression models with a random effect per participant. The primary effect of interest was of a time–medication use interaction term that was used to compare the annualized rate of worsening between the β-blocker and non–β-blocker users over time. These models also were doubly robust and contained all covariates that participants were matched on, as well as a baseline age CAG interaction term. Three models were constructed to investigate the rate of change of the TMS, TFC, and SDMT. Results were false discovery rate–corrected to account for multiple comparisons.^19^

#### Post Hoc Analyses

We performed 3 post hoc analyses. First, we repeated the primary analyses but split β-blocker users into groups based on whether they were using β1-receptor–selective medication or a nonselective β-blocker. Second, we evaluated the effect of ACEI/ARBs on the timing of motor diagnosis and on the rate of symptom progression. Third, we evaluated the effect of β-blockers on anxiety reduction and how that may have impacted the primary results. The methods used for 3 post hoc analyses are described in the eMethods in Supplement 1.

#### Standard Protocol Approvals, Registrations, and Patient Consents

All Enroll-HD sites were required to obtain and maintain local Ethics Committee approvals. Participants must have signed informed consent forms for their data to be included in the datasets.^20^

#### Data Availability

These results were generated using the Enroll-HD database,^20^ which is funded by the CHDI Foundation. This dataset is made available to any interested researcher working at a recognized research institution through a straightforward data use agreement approval process.

## Results

### Survival Analysis Assessing Effect of β-Blocker on AO in Participants With preHD

**Figure 1.**
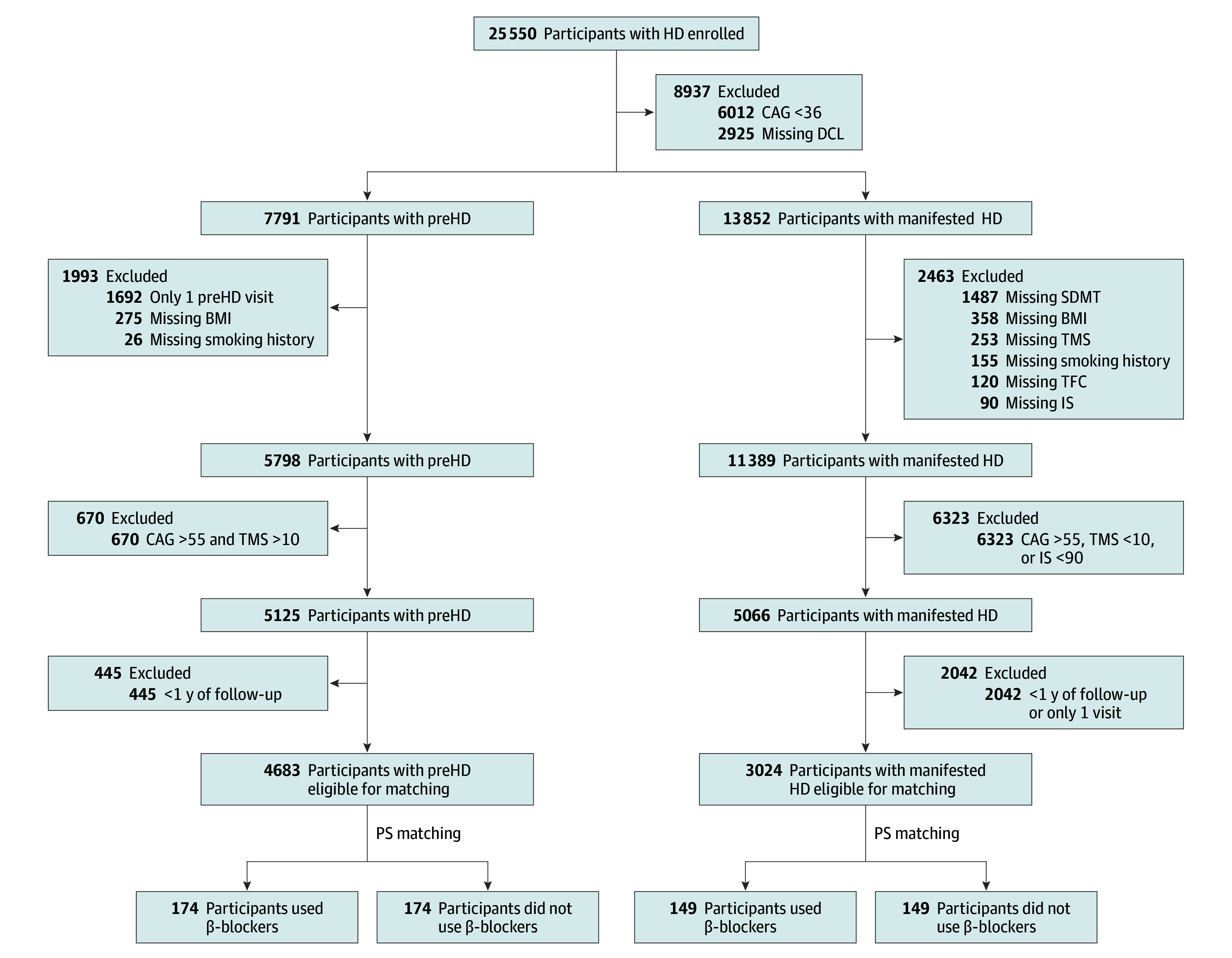
Inclusion and Exclusion Criteria Flowchart showing patients with premanifest Huntington disease (preHD) or manifested Huntington disease (HD) who were taking a β-blocker or were eligible for matching. BMI, indicates body mass index; CAG, cytosine-adenine guanine; DCL, diagnostic confidence level; IS, independence scale; PS, propensity score; SDMT, symbol digit modalities test; TFC, total functional capacity; TMS, total motor score.

**Figure 2.**
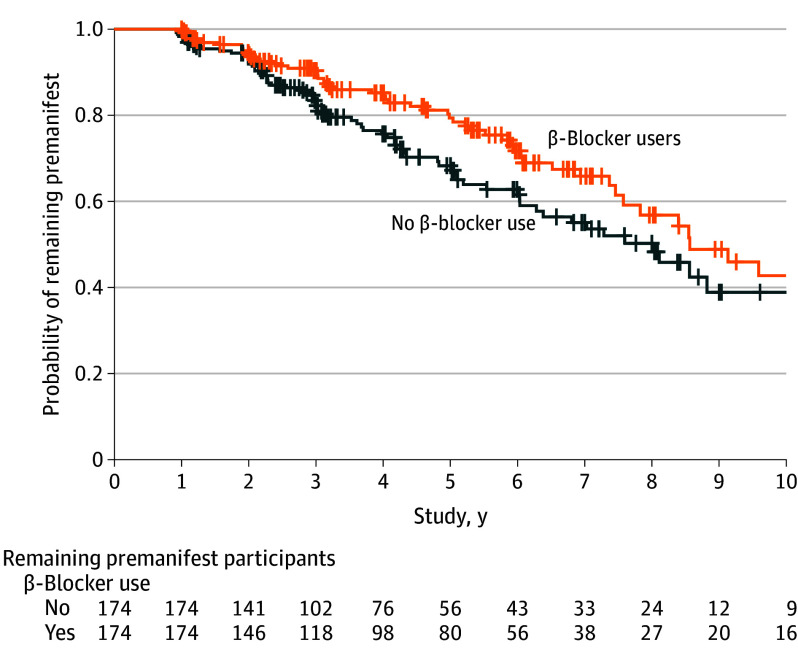
Survival Analysis of Premanifest Huntington Disease β-Blocker Users vs Nonusers Results of the survival analysis in participants with premanifest Huntington disease indicating that participants using a β-blocker had a significantly lower annualized hazard of receiving a motor diagnosis compared with matched participants who were not using a β-blocker.

Of 4683 eligible participants with preHD, 174 were β-blocker users (Figure 1) who were well matched to nonusers (Table). Specifically, the mean baseline age of β-blocker users was 46.4 (SD, 13.1) years vs 48.1 (SD, 12.9) years among nonusers. The mean CAG repeat length among β-blocker users was 41.1 (SD, 2.4) compared with 40.9 (SD, 2.3) in the non–β-blocker users. The most commonly used β-blocker was propranolol (n = 59), followed by metoprolol (n = 56), bisoprolol (n = 36), nebivolol (n = 9), carvedilol (n = 4), atenolol (n = 4), betaxolol (n = 3), sotalol (n = 1), labetalol (n = 1), and nadolol (n = 1). The mean number of years participants were using a β-blocker in the preHD group was 6.93 (SD, 6.18) years. Hypertension was the most common indication for β-blocker use (n = 78 [44.8%]), followed by depression and anxiety (n = 24 [13.8%]) and arrhythmias (n = 22 [12.6%]; eTable 1 in Supplement 1). Importantly, β-blocker users and nonusers were well matched based on potentially confounding cardiovascular comorbidities. Specifically, 79 β-blocker users had a diagnosis of hypertension (45.4%) compared with 80 non–β-blocker users (46.0%), 14 β-blocker users had a diagnosis of diabetes (8.0%) compared with 14 nonusers (8.0%), and 50 β-blocker users were taking a statin (28.7%) compared with 42 of the non–β-blocker users (24.1%) (Table). The β-blocker users had a significantly decreased annualized hazard of receiving a motor diagnosis compared with the β-blocker nonusers (hazard ratio [HR], 0.57; 95% CI, 0.46-0.94; *P* = .02; Figure 2). Participants were compared taking 1 of the 3 most commonly used β-blockers (propranolol, metoprolol, and bisoprolol) to the nonusers. The annualized hazard of receiving a motor diagnosis was decreased 38% among the propranolol users (HR, 0.62; 95% CI, 0.36-1.08; *P* = .09), 19% among metoprolol users (HR, 0.81; 95% CI; 0.49-1.35; *P* = .42), and 34% among bisoprolol users (HR, 0.66; 95% CI, 0.36-1.22; *P* = .19).

**Table.** Baseline Demographics

Characteristic	No. (%)					
	Premotor-manifest HD			Motor-manifest HD		
	β-Blocker		*P* value	β-Blocker		*P* value
	Users	Nonusers		Users	Nonusers	
No.	174	174	NA	149	149	NA
Age, mean (SD)	46.4 (13.1)	48.1 (12.9)	.24	58.9 (11.3)	59.4 (10.6)	.65
Sex						
Female	115 (66.1)	115 (66.1)	>.99	63 (42.3)	58 (38.9)	.64
Male	59 (33.9)	59 (33.9)		86 (57.7)	91 (61.1)	
Race and ethnicity^a^						
Asian	0	0	.50	0	1 (0.7)	.57
Black/African American	0	0		0	1 (0.7)	
Hispanic/Latinx	0	3 (1.7)		4 (2.7)	2 (1.3)	
Multiracial	1 (0.0)	1 (0.6)		0	0	
White	169 (97.1)	168 (96.6)		141 (94.6)	141 (94.6)	
Tobacco history	92 (52.9)	87 (50.0)	.67	76 (51.0)	78 (52.3)	.91
Hypertension	79 (45.4)	80 (46.0)	>.99	91 (61.1)	88 (59.1)	.81
Diabetes	14 (8.0)	14 (8.0)	>.99	14 (9.4)	13 (8.7)	>.99
Statin	50 (28.7)	42 (24.1)	.40	56 (37.6)	59 (39.6)	.81
CAG, mean (SD)	41.1 (2.4)	40.9 (2.3)	.36	42.0 (2.3)	41.9 (2.1)	.71
ACEI/ARB	56 (32.2)	52 (29.9)	.73	68 (45.6)	57 (38.3)	.24
Thiazide	20 (11.5)	13 (7.5)	.27	8 (5.4)	11 (7.4)	.64
BMI,^b^ mean (SD)	28.8 (5.9)	28.8 (7.4)	.90	26.9 (4.3)	26.8 (5.0)	.86
TMS, mean (SD)	2.6 (2.8)	2.9 (4.6)	.33	24.8 (10.4)	24.3 (9.8)	.63
TFC, mean (SD)	12.6 (1.2)	12.7 (1.7)	.50	11.5 (1.7)	11.6 (1.5)	.67
SDMT, mean (SD)	47.3 (12.3)	47.4 (10.5)	.43	29.6 (11.6)	29.9 (10.7)	.81
Anxiety score, mean (SD)	2.18 (3.04)	2.17 (3.08)	.98	2.16 (3.07)	2.21 (3.13)	.75

Abbreviations: ACEI, angiotensin converting enzyme inhibitor; ARB, angiotensin II receptor blocker; BMI, body mass index; CAG, cytosine-adenine-guanine; HD, Huntington disease; NA, not applicable; SDMT, symbol digit modalities test; TFC, total functional capacity; TMS, total motor score.

^a^ Race and ethnicity were self-reported.

^b^ Calculated as weight in kilograms divided by height in meters squared.

### Longitudinal Analyses Assessing Effect of β-Blocker on Progression in Participants With mmHD

There were 3024 eligible participants with mmHD identified (Figure 1). Of those, there were 149 qualifying β-blocker users. These participants were successfully matched to 149 nonusers (Table). Specifically, the mean baseline age of the β-blocker users was 58.9 (SD, 11.3) years compared with 59.4 (10.6) years for the non–β-blocker users. The mean CAG repeat length among β-blocker users was 42.0 (SD 2.3) vs 41.9 (SD, 2.1) for nonusers. Metoprolol was the most commonly used medication in this group (n = 52), followed by bisoprolol (n = 37), propranolol (n = 24), atenolol (n = 11), carvedilol (n = 11), nebivolol (n = 8), nadolol (n = 3), sotalol (n = 2), and betaxolol (n = 1). The mean length of follow-up among β-blocker users was 4.15 (SD, 2.51) years compared with 5.19 (2.92) years for nonusers. Again, hypertension was the most common indication for β-blocker use (n = 86 [57.7%]), followed by coronary artery disease (n = 26 [17.4%]) and arrhythmias (n = 15 [10.1%]; eTable 2 in Supplement 1). As with the premanifest participants, motor-manifest β-blocker users and nonusers were well matched based on potentially confounding cardiovascular comorbidities. Specifically, 91 β-blocker users had a diagnosis of hypertension (61.1%) compared with 88 non–β-blocker users (59.1%), 14 β-blocker users had a concomitant diagnosis of diabetes (9.4%) compared with 13 nonusers (8.7%), and 56 β-blocker users were also taking a statin (37.6%) compared with 59 of the non–β-blocker users (39.6%) (Table).

**Figure 3.**
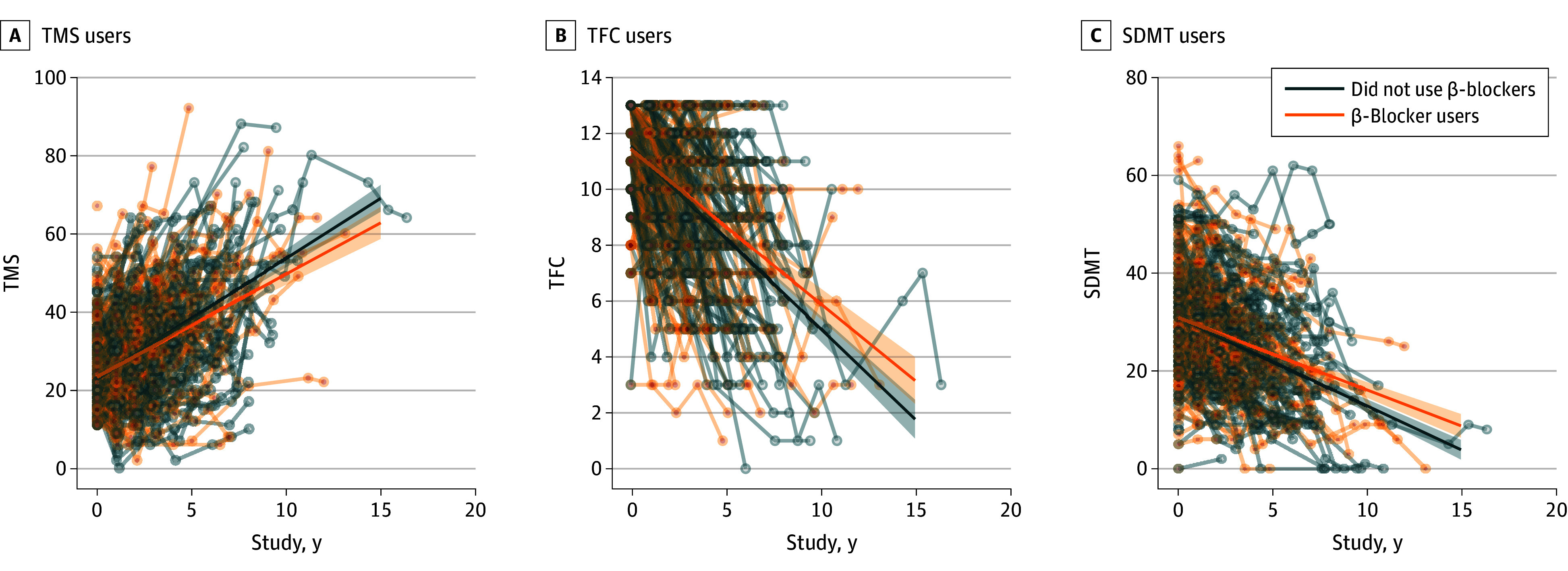
β-Blocker Use in Participants With Motor-Manifest Huntington Disease (mmHD) Was Associated With Slower Rate of Symptom Progression Participants with mmHD who were using a β-blocker had a significantly slower annualized rate of worsening in the total motor score (TMS) (A), total functional capacity (TFC) (B), and symbol digit modalities test (SDMT) (C) compared with matched nonusers. The solid lines represent the predicted regression lines and the shaded area represents the 95% CIs. Thinner lines and points represent data points for individual participants.

The mean annualized rate of change in TMS among β-blocker users was 2.62 points per year compared with 3.07 points per year in the non–β-blocker users (mean difference [MD], −0.45; 95% CI, −0.85 to −0.06; *Q* = 0.025; Figure 3A). The mean annualized rate of decline in the TFC was −0.55 points per year in the β-blocker users compared with −0.65 points per year in the non–β-blocker users (MD, 0.10; 95% CI, 0.02-0.18; *Q* = 0.025; Figure 3B). The mean annualized rate of decline in the SDMT was −1.47 points per year in the β-blocker users compared with −1.80 points per year in the non–β-blocker users (MD, 0.33; 95% CI, 0.10-0.56; *Q* = 0.017; Figure 3C). Again, these analyses were repeated including only the 3 most commonly used β-blockers (propranolol, metoprolol, and bisoprolol). Metoprolol had a significantly slower rate of worsening of the TMS score relative to nonusers (MD, −1.02; 95% CI, −1.70 to 0.33; *P* = .004), but the difference was not significant for propranolol (MD, −0.03; 95% CI, −0.66 to 0.61; *P* = .93) or bisoprolol (−0.51; 95% CI, −1.11 to 0.09; *P* = 1.00). Bisoprolol had a significantly slower rate of worsening of the TFC score relative to nonusers (MD, 0.19; 95% CI, 0.08-0.48; *P* = .002), but the difference was not significant for propranolol (MD, 0.04;95% CI, −0.09 to 0.17; *P* = .55) or metoprolol (MD, −0.55; 95% CI, −0.20 to 0.09; *P* = .45). Similarly, bisoprolol had a significantly slower rate of worsening of the SDMT score relative to nonusers (MD, 0.52; 95% CI, 0.15-0.87; *P* = .01), but the difference was not significant for propranolol (MD, 0.09; 95% CI, −0.28 to 0.47; *P* = .64) or metoprolol (MD, 0.19; 95% CI, −0.22 to 0.60; *P* = .36).

### Post Hoc Analyses: Selective vs Nonselective β-Blocker Use

Both the selective β-blocker users (HR, 0.70; 95% CI, 0.46-1.05; *P* = .08) and nonselective β-blocker users (HR, 0.59; 95% CI, 0.35-1.01; *P* = .05) had a lower annualized hazard of receiving a motor diagnosis relative to the non–β-blocker users, but neither result reached statistical significance (eFigure 1 in Supplement 1). Additionally, there was no significant difference between the selective β-blocker users as compared with the nonselective β-blocker users (HR, 1.18; 95% CI, 0.65-12.14; *P* = .58; eFigure 1 in Supplement 1).

Among the participants with mmHD, selective β-blocker users had a significantly slower mean annualized rate of change in TMS (MD, −0.57; 95% CI, −1.02 to −0.13; *Q* = 0.018) compared with the non–β-blocker users. The selective β-blocker users also had significantly slower mean annualized rate of change in TFC score (MD, 0.10; 95% CI, 0.01-0.19; *Q* = 0.032) and SDMT score (MD, 0.41; 95% CI, 0.15-0.68; *Q* = 0.009). In contrast, nonselective β-blocker users did not differ significantly from the non–β-blocker users in their trajectory of change of TMS (*Q* = 0.493), TFC (*Q* = 0.369), or SDMT (*Q* = 0.493; eTable 3 in Supplement 1). The selective β-blocker users had slower annualized rates of progression of the TMS, TFC, and SDMT compared with the nonselective users, but the results did not reach statistical significance (*Q* = 0.386, *Q* = 0.923, and *Q* = 0.336, respectively; eTable 3 in Supplement 1).

### Post Hoc Analyses: ACEI and ARB Users

There were 186 participants with preHD using an ACEI or an ARB who were matched to 186 participants with preHD not using an ACEI/ARB (eTable 4 in Supplement 1). The use of an ACEI/ARB was not associated with a significant decrease in the annualized risk of receiving a motor diagnosis of HD relative to the non–ACEI/ARB users (HR, 0.81; 95% CI, 0.58-1.14; *P* = .23; eFigure 2 in Supplement 1).

There were 193 patients with early mmHD using an ACEI/ARB who were matched to 193 non–ACEI/ARB users with mmHD (eTable 4 in Supplement 1). The ACEI/ARB users did not differ from the nonusers in annualized rate of change of TMS, TFC, or SDMT (eTable 5 in Supplement 1).

### Post Hoc Analyses: Effect of β-Blocker-Induced Anxiety Reduction

There was no significant difference in baseline anxiety scores between the preHD β-blocker users (2.18 [SD, 3.04]) and nonusers (2.17 [SD, 3.08]). Furthermore, there was no significant difference in the mean change from baseline of anxiety score at the final visit between β-blocker users (0.25 [SD, 3.17]) and nonusers (0.28 [SD, 3.78]).

Among the participants with mmHD, there was no significant difference in the rate of progression of anxiety symptoms between β-blocker users and nonusers (MD, −0.02; 95% CI, −0.08 to 0.43; *P* = .55). Furthermore, there were no significant correlations between the slope of change of anxiety and TMS (*r*^2^ = −0.05; *P* = .52), TFC (*r*^2^ = 0.01; *P* = .91), or SDMT (*r*^2^ = 0.06; *P* = .46) among β-blocker users.

## Discussion

β-Blockers were associated with a significant reduction of the annualized hazard of motor diagnosis in preHD and were associated with slower worsening of symptoms in participants with mmHD. Post hoc analyses revealed that the observed effects of β-blockers on clinical worsening in mmHD may have been primarily driven by antagonism of β-1 receptors. Additional post hoc analyses failed to demonstrate significant beneficial effects of ACEI/ARBs in patients with preHD or mmHD.

β-Blockers may exert their effects in HD by blocking norepinephrine signaling, although the exact mechanism remains unclear. This is based on the differential findings in β-blocker users compared with ACEI/ARB users. Specifically, both ACEI/ARBs and β-blockers impact the autonomic nervous system by decreasing sympathetic tone, which is increased in patients with HD. However, β-blockers do this by blocking the action of norepinephrine at the noradrenergic receptor. ACEI/ARBs decrease sympathetic tone by decreasing norepinephrine release from sympathetic nerves via angiotensin II blockade. Because β-blockers were associated with a decreased annualized risk of receiving a motor diagnosis of HD and slower worsening of symptoms in preHD and participants with mmHD and ACEI/ARBs were not, we hypothesize that noradrenergic transmission may play a role. However, this is beyond the scope of this article and further studies would be required to confirm this hypothesis.

In interpreting the findings of this study, it is crucial to consider the potential symptomatic effects of β-blockers. Notably, substances like propranolol have been shown to alleviate symptoms, such as tardive dyskinesia and anxiety.^21^ Reducing anxiety, in particular, can profoundly affect patient functionality, motor symptoms, and quality of life. The potential for β-blockers to alleviate anxiety, therefore, could indirectly influence the timing of clinical diagnosis of HD and the overall progression metrics of HD. Our post hoc analysis examining the effect of β-blockers on anxiety scores aimed to elucidate this aspect further. Our analyses in participants with preHD and mmHD did not reveal significant improvements in anxiety between the β-blocker users and nonusers. Furthermore, the rate of change in anxiety scores among mmHD β-blockers users was not correlated with the rate of change of clinical outcome measures of HD. However, it is still possible that the observed effects of β-blockers in HD was influenced by β-blockers’ potential to reduce anxiety and improve daily function in patients with HD.

### Limitations

The primary limitation of this study is the fact that all of the results represent associations rather than causative changes induced by β-blockers. Furthermore, these results are unable to determine potential mechanisms by which β-blockers exert their effect in HD. Ideally, a comparison of lipophilic β-blockers, which are more capable of crossing the blood-brain barrier, with hydrophilic β-blockers would provide some insight into whether the primary site of action occurred centrally or peripherally. Unfortunately, an overwhelming majority of preHD β-blockers users in this study were taking lipophilic products (96.5%). This was true of the mmHD β-blocker users, as well (89.3%). As a result, robust analyses comparing the effects between hydrophilic and lipophilic products was not possible, which may limit the interpretability of these results. Similarly, this study did not evaluate dose-related effects of β-blockers. A wide range of β-blockers were represented in the preHD and mmHD users and each individual medication may be used for various indications at differing daily doses. Therefore, attempting to standardize doses across all β-blockers to evaluate the effect of dose may introduce significant confounding. The lack of ability to investigate a dose-related effect may limit the interpretability of these findings. Selection biases may have occurred. β-blocker use is likely more common in older individuals with more cardiovascular comorbidities. An individual with HD who lives long enough to develop some of the age-related comorbidities prompting the use of a β-blocker will likely have fewer CAG repeats. Propensity score matching aims to decrease selection bias in our study, but will not eliminate it. Another selection bias may have been introduced based on health care utilization. Specifically, patients with HD who are taking a β-blocker may represent a group who receives higher-quality health care, or more consistently seeks out health care, relative to non–β-blocker users. If true, our results may not be suggesting that the use of β-blockers is associated with slower worsening of symptoms in patients with HD; rather, they may be indicating that patients with HD who have access to high-quality health care may be able to avoid the detrimental impact that comorbidities, such as hypertension, seem to have on HD. To investigate the potential for this selection bias, we evaluated the participants who were not taking a β-blocker who had a concomitant diagnosis of hypertension (n = 80 in preHD and n = 88 in mmHD; Table). The presence of these participants raises the question of whether or not these participants are less willing or have a decreased ability to treat their hypertension. However, among those 80 non–β-blocker users with preHD and hypertension, 68 were receiving treatment with another class of medication (ACEI/ARB, diuretic, or calcium channel blocker) to treat their blood pressure. Furthermore, 78 of 88 participants with mmHD and hypertension who were not being treated with a β-blocker were being treated with another class of medication. This may suggest that the influence of this selection bias may be minimal, it cannot be ruled out. Lastly, the Enroll-HD dataset does not contain information about heart rate or blood pressure for participants. Without this information, we are limited in our ability to determine the impact that blood pressure and blood pressure control played in this study.

## Conclusions

Overall, we have demonstrated that the use of β-blockers was associated with a significantly lower annualized risk of receiving a clinical diagnosis of HD in participants with preHD; furthermore, β-blockers were associated with a slower rate of worsening of clinical symptoms of HD among participants with mmHD. However, further studies are warranted to elucidate the possible mechanism by which β-blockers may positively influence HD outcomes.

## Notes

Supplement 1. eMethods eTable 1. Indication for Use of Beta-Blockers in preHD Subjects eTable 2. Indication for Use of Beta-Blockers in mmHD Subjects eTable 3. Longitudinal Results in mmHD of Selective vs Non-selective Users eTable 4. ACEI/ARB Users and Non-Users Baseline Demographics eTable 5. ACEI/ARB Users vs Non-Users eFigure 1. Survival analysis of preHD Selective and Non-Selective beta-blocker Users vs. Non-Users eFigure 2. Survival analysis of preHD ACEI/ARB Users vs. Non-Users

Supplement 2. Data sharing statement
