# Supplementary material for: β-Blocker Use and Delayed Onset and Progression of Huntington Disease
Source: JAMA Neurol. 2024 Dec 2;82(1):85–92. doi: 10.1001/jamaneurol.2024.4108 (PMC11612910; doi:10.1001/jamaneurol.2024.4108)
Supplement: Supplement 1. — eMethods eTable 1. Indication for Use of Beta-Blockers in preHD Subjects eTable 2. Indication for Use of Beta-Blockers in mmHD Subjects eTable 3. Longitudinal Results in mmHD of Selective vs Non-selective Users eTable 4. ACEI/ARB Users and Non-Users Baseline Demographics eTable 5. ACEI/ARB Users vs Non-Users eFigure 1. Survival analysis of preHD Selective and Non-Selective beta-blocker Users vs. Non-Users eFigure 2. Survival analysis of preHD ACEI/ARB Users vs. Non-Users [file jamaneurol-e244108-s001.pdf]

Supplemental Online Content

Schultz JL, Ogilvie AC, Harshman LA, Nopoulos PC. Beta-blocker use and delayed onset and progression of Huntington's disease. *JAMA Neurol*. Published online December 2, 2024. doi:10.1001/jamaneurol.2024.4108

eMethods
eTable 1. Indication for Use of Beta-Blockers in preHD Subjects
eTable 2. Indication for Use of Beta-Blockers in mmHD Subjects
eTable 3. Longitudinal Results in mmHD of Selective vs Non-selective Users
eTable 4. ACEI/ARB Users and Non-Users Baseline Demographics
eTable 5. ACEI/ARB Users vs Non-Users
eFigure 1. Survival analysis of preHD Selective and Non-Selective beta-blocker Users vs. Non-Users
eFigure 2. Survival analysis of preHD ACEI/ARB Users vs. Non-Users

This supplemental material has been provided by the authors to give readers additional information about their work.

© 2024 Schultz JL et al. *JAMA Neurol.*

eMethods-Clinical Outcome Measures

HD-ISS

Patients in the last stage of disease (stage 3), per the HD-ISS, are defined as having signs or symptoms of disease and functional changes based on the UHDRS Total Functional Capacity (TFC) and the Independence Scale. Patients within stage 3 are further divided into mild, moderate, or severe disease. Mild disease is defined as participants not requiring assistance with routine activities, although the activities may be difficult to perform or take a long time. We used the UHDRS Independence Scale to help distinguish such subjects with early mmHD.

Independence Scale

The UHDRS Independence Scale measures a subject's functional ability, including completion of activities of daily living.[1] It ranges from 100% down to 0% with lower values indicating worsening functionality and independence. Patients with ≥ 90% on the Independence Scale have no physical care requirements if difficult tasks are avoided. In contrast, patients with an 80% have a change in their pre-disease level of employment and cannot perform household chores to their pre-disease level and may need help with their finances. Based on this, participants in the mmHD group had to have a baseline UHDRS Independence Scale score ≥ 90% to ensure that they were early in their disease.

Total Motor Score

The UHDRS TMS has a range of scores from zero to 124. There are 31 scales that assess various aspects of a subject's movements, including ocular movements, chorea, bradykinesia, gait, dystonia, and rigidity, among other areas. Higher scores are associated with worsening motor symptoms.[1]

© 2024 Schultz JL et al. *JAMA Neurol*.

Total Functional Capacity

The TFC measures a subject's ability to continue to perform activities, including continuation of gainful employment, managing one's finances, performing everyday chores, and remaining in an independent care setting. The TFC score can range from a maximum score of 13, indicating higher function, to a minimum score of 0, indicating lower functionality.[1]

Symbol Digit Modalities Test

The SDMT is commonly used in research settings to assess neurological dysfunction with a particular focus on attention, perceptual speed, motor speed, and visual scanning. The SDMT is sensitive to changes in cognitive function over time, making it an ideal candidate for monitoring longitudinal progression.[2]

Defining β-Blocker Users

Participants using ophthalmologic, timolol-based products were excluded from these analyses due to the inconsistent and relatively low systemic absorption of timolol eye drops. Also, participants who were using acebutolol or pindolol were excluded given their intrinsic sympathomimetic activity.[3]

If a preHD subject had at least a year of qualifying β-blocker use and then discontinued treatment prior to their event of interest, they were still considered a β-blocker user. Participants were considered to have uninterrupted β-blocker use if they did not have a break in therapy for more than 90 days. Some participants had uninterrupted use but switched from one β-blocker to another. In this instance, we report the medication the participant was taking for the longest period. If a participant had a break of more than 90 days in β-blocker therapy and then resumed treatment

© 2024 Schultz JL et al. *JAMA Neurol*.

with a β-blocker, the longest of any qualifying blocks of time of β-blocker use was utilized for these analyses.

Post-Hoc Analyses: Methods

1. *Selective versus Non-Selective* β-*Blocker Use*

   After performing the primary survival and longitudinal analyses, we investigated differences between participants using a β1-receptor-selective medication versus participants who were using a non-selective β-blocker. The selective β-blockers were atenolol, betaxolol, bisoprolol, metoprolol, and nebivolol. Non-selective medications included carvedilol, labetalol, nadolol, propranolol, and sotalol. We performed a similar survival analysis in the preHD individuals and constructed similar LMER models in the mmHD individuals.

2. *ACEI and ARB Users*

   Hypertension is associated with a significantly elevated risk of receiving a motor diagnosis of HD.[4,5] However, the use of antihypertensive medications has also been shown to improve clinical outcome measures in HD.[5] Therefore, we investigated the association between the use of ACEI/ARBs with the annualized risk of receiving a motor diagnosis in preHD individuals and the rate of clinical progression in mmHD individuals. ACEI/ARBs were chosen because they are considered a first-line treatment for hypertension and were more commonly prescribed to patients with HD in the Enroll-HD study. In contrast, thiazide diuretics are a commonly prescribed first-line antihypertensive, but there were substantially fewer users of these medications within the Enroll-HD database (Table 1).

© 2024 Schultz JL et al. *JAMA Neurol.*

For post-hoc analyses assessing ACEI/ARBs, users were those participants with a reported use of a medication with WHO Anatomical Therapeutic Chemical Index numbers of C09AA or C09CA. All other criteria for inclusion were similar to those used to define the use of β-blockers for preHD and mmHD subjects.

3. *Effect of β-blocker-Induced Anxiety Reduction*

β-blockers can be used to reduce anxiety, which is a common symptom of HD.[6] Furthermore, worsening anxiety can have a negative impact on clinical symptoms of HD, including motor score.[7] As a result, the β-blocker users could be hypothesized to have improved clinical outcomes as a result of improved anxiety. To evaluate this potential confounder, we quantified differences in anxiety symptoms at baseline between β-blocker users and non-users in the preHD and mmHD subjects. Anxiety was quantified using the Problem Behaviors Assessment (PBA) for HD.[8] Specifically, the product of the anxiety severity (possible values of 0 – 4) subscale and the anxiety frequency (possible values of 0 – 4) was used to assess anxiety amongst all subjects. For the preHD subjects, we then compared the mean change from baseline in anxiety scores between groups. For the mmHD subjects, we aimed to determine if there was a significant effect of β-blockers on the progression of anxiety scores. This was done by repeating the linear mixed-effects regression analyses that were used to evaluate TMS, TFC, and SDMT but the anxiety score was the dependent variable. Next, to determine if changes in anxiety scores correlated with clinical progression of symptoms of HD amongst mmHD β-blocker users, we also extracted each subject's slope of change of the TMS, TFC, and SDMT as well as their anxiety score. We then correlated the slope of change of the calculated anxiety score with the slope of change of each clinical symptom.

© 2024 Schultz JL et al. *JAMA Neurol.*

eTable 1 – Indication for Use of Beta-Blockers in preHD Subjects

	Arrhythmia	CAD	Depression & Anxiety	HTN	Migraine	Tremor	Other	**Total**
Propranolol	3	0	24	9	12	8	3	**59**
Metoprolol	10	5	0	33	5	0	3	**56**
Bisoprolol	6	6	0	21	2	0	1	**36**
Nebivolol	0	1	0	7	1	0	0	**9**
Carvedilol	0	1	0	3	0	0	0	**4**
Atenolol	0	0	0	3	1	0	0	**4**
Betaxolol	1	0	0	1	0	1	0	**3**
Sotalol	1	0	0	0	0	0	0	**1**
Labetalol	0	0	0	1	0	0	0	**1**
Nadolol	1	0	0	0	0	0	0	**1**
**Total**	**22**	**13**	**24**	**78**	**21**	**9**	**7**	**174**

CAD – Coronary Artery Disease
HTN - Hypertension

© 2024 Schultz JL et al. *JAMA Neurol.*

eTable 2 - Indication for Use of Beta-Blockers in mmHD Subjects

	Arrhythmia	CAD	Depression & Anxiety	HTN	Migraine	Tremor	Other	**Total**
Propranolol	1	0	10	4	8	1	0	**24**
Metoprolol	7	7	0	37	0	0	1	**52**
Bisoprolol	3	9	0	25	0	0	0	**37**
Nebivolol	1	1	0	6	0	0	0	**8**
Carvedilol	0	7	0	3	0	0	1	**11**
Atenolol	0	2	0	9	0	0	0	**11**
Betaxolol	0	0	0	1	0	0	0	**1**
Sotalol	2	0	0	0	0	0	0	**2**
Nadolol	1	0	0	1	1	0	0	**3**
**Total**	**15**	**26**	**10**	**86**	**9**	**1**	**2**	**149**

CAD – Coronary Artery Disease
HTN - Hypertension

© 2024 Schultz JL et al. *JAMA Neurol.*

eTable 3 – Longitudinal Results in mmHD of Selective vs Non-selective Users

	Non-Users		Selective Users		Non-Selective Users	
	Mean Δ	95% CI	Mean Δ	95% CI	Mean Δ	95% CI
TMS	3.07	2.82 – 3.33	2.50*	1.98 – 3.01	2.87	2.23 – 3.50
TFC	-0.65	-0.70 – -0.60	-0.55*	-0.66 – -0.45	-0.56	-0.69 – -0.43
SDMT	-1.80	-1.95 – -1.65	-1.39*	-1.69 – -1.08	-1.64	-2.02 – -1.27

*Statistically significant difference compared to Non-Users

CI: Confidence Interval
SDMT: Symbol Digit Modalities Test
TFC: Total Functional Capacity
TMS: Total Motor Score

© 2024 Schultz JL et al. *JAMA Neurol.*

eTable 4 – ACEI/ARB Users and Non-Users Baseline Demographics

	Pre-Motor-Manifest HD			Motor Manifest HD		
	ACEI/ARB Users	ACEI/ARB Nonusers	p-value	ACEI/ARB Users	ACEI/ARB Nonusers	p-value
N	186	186	N/A	193	193	N/A
Age, mean ± S.D.	49.8 ± 10.6	50.1 ± 11.8	0.817	59.1 ± 10.8	60.1 ± 11.0	0.388
% Male, n (%)	86 (46.2)	81 (43.5)	0.677	117 (60.6)	122 (63.2)	0.675
% Tobacco Hx, n (%)	94 (50.5)	82 (44.1)	0.253	97 (50.3)	103 (53.4)	0.611
% HTN, n (%)	147 (79.0)	150 (80.6)	0.796	149 (77/2)	146 (75.6)	0.810
% DM, n (%)	24 (12.9)	21 (11.3)	0.750	22 (11.4)	23 (11.9)	1.000
% Statin, n (%)	60 (32.3)	56 (30.1)	0.737	70 (36.3)	71 (36.8)	1.000
% β-blocker , n (%)	43 (23.1)	28 (15.1)	0.065	41 (21.2)	26 (13.5)	0.060
CAG, mean ± S.D.	40.9 ± 1.9	41.0 ± 2.0	0.513	41.9 ± 2.1	41.6 ± 2.1	0.213
BMI, mean ± S.D.	29.7 ± 6.1	29.3 ± 6.3	0.460	27.1 ± 5.0	26.7 ± 4.3	0.435
TMS, mean ± S.D.	2.6 ± 2.9	3.2 ± 3.1	0.046	25.1 ± 10.5	25.7 ± 10.6	0.546
TFC, mean ± S.D.	12.6 ± 1.0	12.6 ± 1.2	0.529	11.6 ± 1.6	11.4 ± 1.7	0.330
SDMT, mean ± S.D.	45.8 ± 11.5	45.7 ± 11.5	0.958	29.3 ± 11.3	28.8 ± 11.5	0.632

BMI: Body Mass Index
CAG: Cytosine-Adenine-Guanine
DM: Diabetes Mellitus
HD: Huntington's Disease
HTN: Hypertension
Hx: History
N: Number
S.D.: Standard Deviation
SDMT: Symbol Digit Modalities Test
TFC: Total Functional Capacity
TMS: Total Motor Score

© 2024 Schultz JL et al. *JAMA Neurol.*

eTable 5 – ACEI/ARB Users vs Non-Users

Variable	Mean Δ in ACEI/ARB Users	Mean Δ in ACEI/ARB Non-Users	Group Difference	95% CI	q-value
TMS	2.81	2.28	0.53	-1.21 – 2.27	0.828
TFC	-0.68	-0.53	-0.14	-0.48 – 0.20	0.828
SDMT	-1.73	-1.61	-1.18 – 0.95	-1.95 – -1.65	0.836`

ACEI: Angiotensin Converting Enzyme Inhibitor
ARB: Angiotensin II Receptor Blocker
SDMT: Symbol Digit Modalities Test
TFC: Total Functional Capacity
TMS: Total Motor Score

© 2024 Schultz JL et al. *JAMA Neurol.*

eFigure 1 – Survival analysis of preHD Selective and Non-Selective β-blocker Users vs. Non-Users

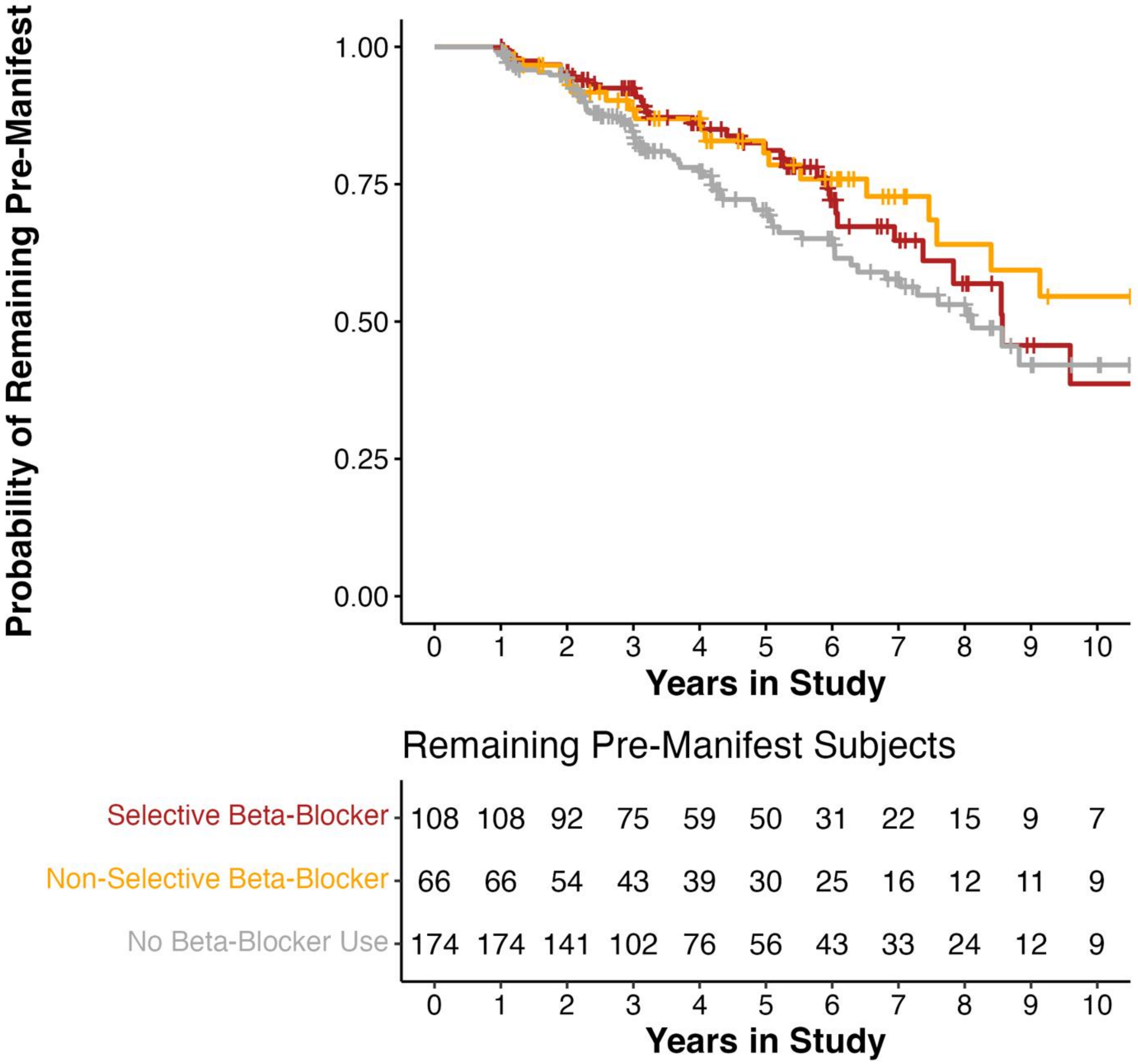

Participants with preHD who were using a selective β-blocker (red line) or a non-selective β-blocker (orange line) had slower annualized hazards of receiving a motor diagnosis compared to participants who were not using a and matched participants not using a β-blocker (gray line), but the results for the individual groups did not reach statistical significance.

HD: Huntington's Disease
SDMT: Symbol Digit Modalities Test (Total Correct)
TFC: Total Functional Capacity
TMS: Total Motor Score

© 2024 Schultz JL et al. *JAMA Neurol.*

eFigure 2 – Survival analysis of preHD ACEI/ARB Users vs. Non-Users

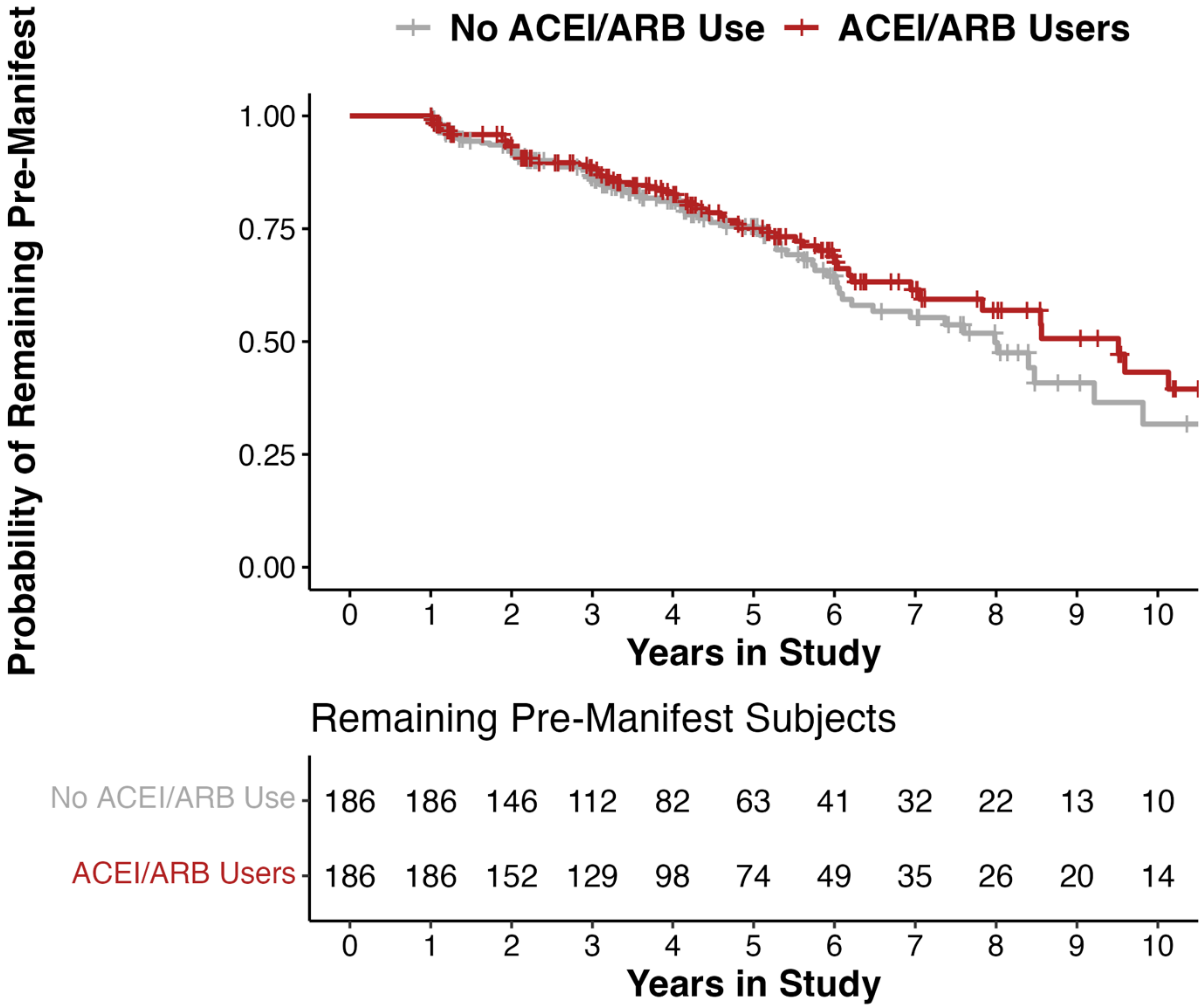

Participants with preHD who were using an ACEI/ARB (red line) did not have a significantly different annualized hazard of receiving a motor diagnosis compared to matched participants who were not using an ACEI/ARB (gray line).

ACEI: Angiotensin Converting Enzyme Inhibitor
ARB: Angiotensin II Receptor Blocker
HD: Huntington's Disease
SDMT: Symbol Digit Modalities Test (Total Correct)
TFC: Total Functional Capacity
TMS: Total Motor Score

© 2024 Schultz JL et al. *JAMA Neurol.*

Supplemental References

1. Unified Huntington's Disease Rating Scale: reliability and consistency. Huntington Study Group. *Mov Disord*. Mar 1996;11(2):136-42. doi:10.1002/mds.870110204
2. Kiely KM, Butterworth P, Watson N, Wooden M. The Symbol Digit Modalities Test: Normative data from a large nationally representative sample of Australians. *Arch Clin Neuropsychol*. Dec 2014;29(8):767-75. doi:10.1093/arclin/acu055
3. Jaillon P. Relevance of intrinsic sympathomimetic activity for beta blockers. *Am J Cardiol*. Sep 25 1990;66(9):21C-23C. doi:10.1016/0002-9149(90)90758-s
4. Schultz JL, Harshman LA, Langbehn DR, Nopoulos PC. Hypertension Is Associated With an Earlier Age of Onset of Huntington's Disease. *Mov Disord*. Sep 2020;35(9):1558-1564. doi:10.1002/mds.28062
5. Steventon JJ, Rosser AE, Hart E, Murphy K. Hypertension, Antihypertensive Use and the Delayed-Onset of Huntington's Disease. *Mov Disord*. Jun 2020;35(6):937-946. doi:10.1002/mds.27976
6. Dale M, van Duijn E. Anxiety in Huntington's Disease. *J Neuropsychiatry Clin Neurosci*. Fall 2015;27(4):262-71. doi:10.1176/appi.neuropsych.14100265
7. Anderson KE, van Duijn E, Craufurd D, et al. Clinical Management of Neuropsychiatric Symptoms of Huntington Disease: Expert-Based Consensus Guidelines on Agitation, Anxiety, Apathy, Psychosis and Sleep Disorders. *J Huntingtons Dis*. 2018;7(3):355-366. doi:10.3233/JHD-180293
8. Craufurd D, Thompson JC, Snowden JS. Behavioral changes in Huntington Disease. *Neuropsychiatry Neuropsychol Behav Neurol*. Oct-Dec 2001;14(4):219-26.

© 2024 Schultz JL et al. *JAMA Neurol.*
