# Supplementary material for: β-Blocker Use and Delayed Onset and Progression of Huntington Disease
Source: JAMA Neurol. 2024 Dec 2;82(1):85–92. doi: 10.1001/jamaneurol.2024.4108 (PMC11612910; doi:10.1001/jamaneurol.2024.4108)
Supplement: Supplement 2. — Data sharing statement [file jamaneurol-e244108-s002.pdf]

Data Sharing Statement

Data
Data available: Yes
Data types: Data dictionary, Deidentified participant data
How to access data: enroll-hd.org
When available: With publication

Supporting Documents
Document types: None

Additional Information
Who can access the data: These results were generated using the Enroll-HD database (enroll-hd.org), which is funded by CHDI, Inc. This dataset is made available to any interested researcher working at a recognized research institution through a straightforward Data Use Agreement approval process.
Types of analyses: Code may be may available upon reasonable request from qualified researchers.
Mechanisms of data availability: Data will be made available related to code with investigator support.
